# Spatial characteristics of soil enzyme activity and bacterial diversity in Chinese egret habitat

**DOI:** 10.1128/spectrum.00015-25

**Published:** 2025-12-30

**Authors:** Mingzhao Han, Ying Han, Xin Liu, Xiao Qin, Jingyang Sun, Peng Li

**Affiliations:** 1 Ministry of Education Key Laboratory for Ecology of Tropical Islands, Key Laboratory of Tropical Animal and Plant Ecology of Hainan Province, College of Life Sciences, Hainan Normal University 117783, Haikou, China; 2 Key Laboratory of Earth Surface Processes and Environmental Change of Tropical Islands, College of Geography and Environmental Science, Hainan Normal University 12389, Haikou, China; University of Mississippi, University, Mississippi, USA

**Keywords:** egret, soil enzyme, microbial diversity, uric acid, island ecosystem

## Abstract

The egret activities, including the deposition of feces and feathers, significantly alter soil properties and the local ecosystem. Two villages of Hainan Province serve as “the haven of egret,” a crucial habitat along the migratory route of egrets in China, where tens of thousands of egrets flock to breed. To explore the spatial dynamics of soil bacterial diversity in egret nesting areas, soil samples of three layers (0–10 cm, 10–20 cm, and 20–30 cm) were collected from five long-term nesting sites. Soil enzyme activity, microbial community composition, and their interaction network were analyzed. Additionally, functional bacteria capable of degrading uric acid were isolated from the surface soil (0–10 cm) to obtain novel strains. The results revealed that soil enzyme activities, including soil urease, acid phosphatase, and sucrase, decreased significantly with increasing soil depth. Among the bacterial communities, Acidobacteriota and Proteobacteria were the dominant microbial phyla (>40%), with Chloroflexi showing increasing abundance with depth, while Verrucomicrobiota and Bacteroidota decreased. Surface soil (0–10 cm) demonstrated significantly higher bacterial alpha and beta diversity than deeper layers (20–30 cm); notably, the Shannon index exhibited a positive correlation with soil pH, urease, and sucrase activities, highlighting the strong association between bacterial diversity and soil properties. Furthermore, five highly effective uric acid-degrading bacterial strains belonging to *Cellulosimicrobium* sp., *Paracoccus* sp., *Arthrobacter* sp. (two strains), and *Paenibacillus* sp. were identified, which highlights their potential in ecological and economic applications. These findings enhance our understanding of soil bacterial community characteristics in egret colonies and provide a foundation for the habitat protection of egrets and island ecosystems.

**IMPORTANCE:**

Egret colony activities have a significant impact on soil bacterial community structure, which is essential for maintaining soil nutrient dynamics and ecological functions. Understanding the spatial distribution characteristics of bacterial communities is fundamental to effective ecological monitoring and conservation. Both alpha and beta diversity exhibited significant variations across different soil layers and showed a strong positive correlation with soil enzyme activity, indicating that soil bacterial diversity is a key driver of nutrient efficiency.

## INTRODUCTION

Overgrazing affects soil nutrient cycling by altering belowground carbon inputs and adding fecal nutrients due to excessive livestock populations, which can threaten the ecosystem balances (1). A study also demonstrated that red fire ant invasions have a significant impact on the structure and function of the soil microbial community (2). Similarly, bird nesting colonies deposit large amounts of allochthonous substances from coastal or forest areas, which affect soil geochemistry and microbial community, ultimately influencing plant growth and the local ecosystem health (3–5). For example, long-term nutrient deposition from Gray Heron colony results in lower pH and significant concentrations of phosphorus (10-fold) and nitrogen (twofold) compared to the surrounding forest (6). Several studies also demonstrated that soil degradation, salinization, and eutrophication commonly occur in areas with bird roosting due to nutrient accumulation (7, 8).

Soil microorganisms play a pivotal role in driving various physicochemical processes, including carbon and nutrient cycling, and they interact closely with soil properties (9). The structure and composition of microbial communities directly influence soil biogeochemistry and enzyme activity, thereby regulating soil quality and promoting plant health (10, 11). Consequently, the soil microbiome is widely recognized as a sensitive indicator of changes within global ecosystems. Interestingly, soil microbial communities are largely affected by factors such as soil pH and total phosphorus levels (12), while birds contribute to the return of phosphorus to terrestrial ecosystems through feces and food debris and reduce soil pH (6, 8, 13). These alterations triggered corresponding changes in soil microbiome, fostering the development of unique bird-seeded vegetation and plant communities (14).

The villages of Danzhou city of Hainan Province, two important breeding habitats for Chinese egrets and other waterbirds in China, host over 5,000 egrets annually (15). These large waterbirds, as apex predators, significantly influence soil microbial profiles through their feeding and nesting activities. While previous studies have primarily focused on the impacts of bird colonies on soil physicochemical properties, heavy metal pollution, and surrounding vegetation, research addressing spatial-scale variations in soil enzyme activity and microbial community diversity remains limited. Birds excrete nitrogenous waste primarily as urate salts and uric acid (16), substances that can serve as indicators of water pollution (17), and are also known to damage property through their corrosive effects. Therefore, this study aims to explore the spatial distribution patterns of soil enzyme activities and microbial diversity associated with the egret nesting colony, examine their interrelationships, and identify specific functional microbes capable of utilizing uric acid. The findings are expected to elucidate the regulatory mechanisms by which egret habitat activities influence soil processes within ecosystems, providing fundamental insights into bird habitat conservation and soil ecology research. Additionally, this study proposes a potential microbe-based approach to mitigate uric acid-induced erosion.

## MATERIALS AND METHODS

### Study area and sampling locations

**Fig 1:**
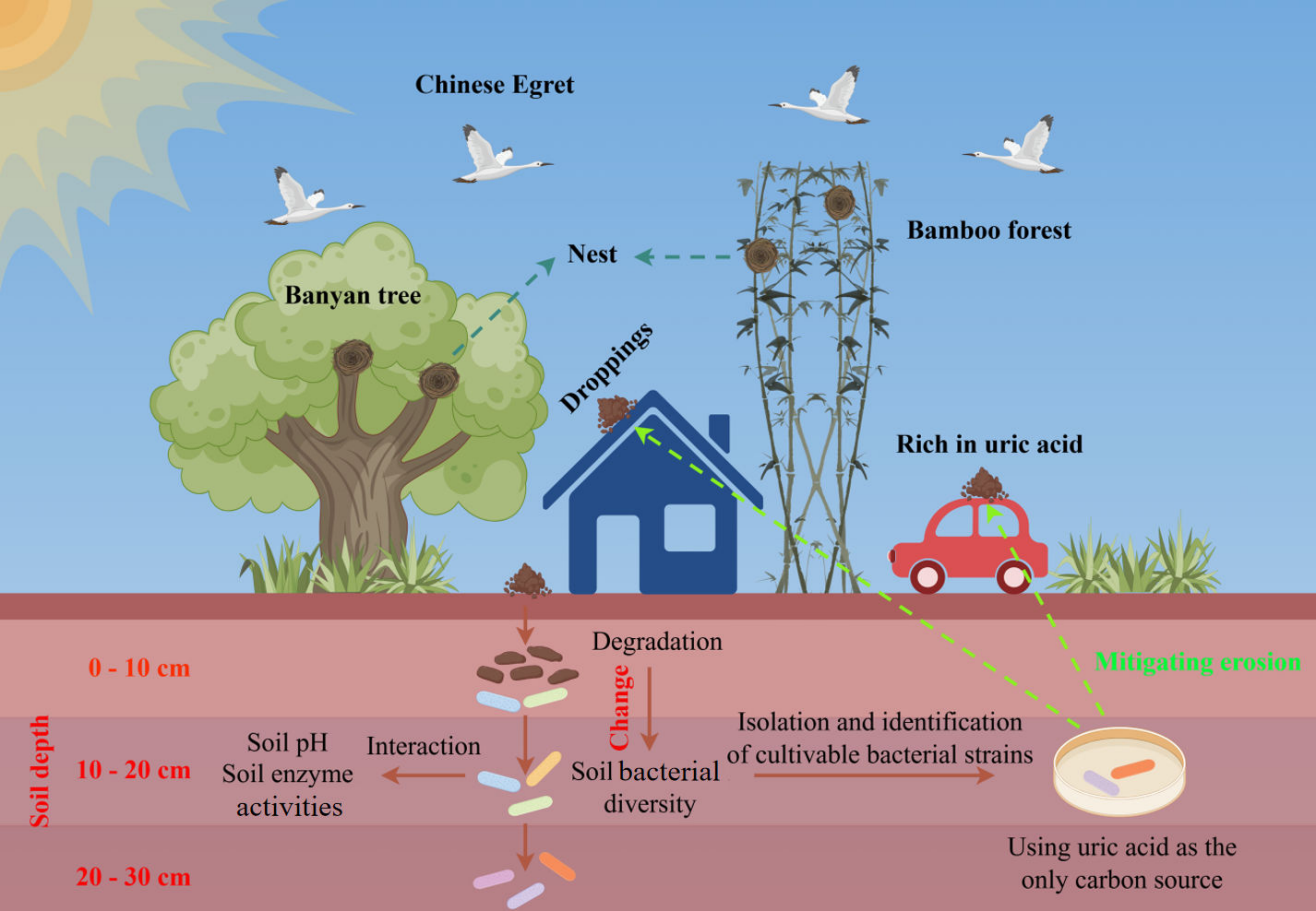
Schematic diagram of the study design and sampling strategy in the Chinese egret colony. This figure illustrates the overall workflow of the study, including: (i) fecal deposition from nesting egrets onto soils beneath host trees; (ii) stratified soil sampling at three depth layers; (iii) analysis of soil enzyme activities, pH value, and bacterial community composition based on 16S rRNA sequencing; and (iv) isolation of uric acid-utilizing bacteria using a selective medium with uric acid as the sole carbon source.

The studied sites were located in Wuji village (19°64′ N, 109°53′ E) and Xinying village (19°48′ N, 109°46′ E), Danzhou City, Hainan Province, China. Hainan’s climate and wetland ecosystems provide ideal conditions for egrets to inhabit and forage, leading them to roost here from October to March—a seasonal pattern that has persisted for thousands of years. Soil samples were collected from banyan trees (*Feng shui* wood) and bamboo forests with nesting colonies. These two vegetation types represent the primary nesting habitats for egret colonies in the region; thus, our sampling design was intended to reflect this ecological reality. Five sampling sites were selected in total: two locations in Wuji Village (WF1 and WF2, both under banyan trees) and three in Xinying Village (XF1 under banyan trees; XB1 and XB2 in bamboo forest). Undisturbed soil (0–30 cm) was sampled at depths of 0–30 cm using a soil collector, with three soil layers segmented into 0–10 cm (A), 10–20 cm (B), and 20–30 cm (C) sections. Samples were stored in sterile plastic bags, kept in an icebox, and immediately transported to the laboratory. Then, the stones and plant residues were removed; one portion of each sample was air-dried for soil pH and enzyme activity analyses, while another portion was stored at −80℃ until subsequent 16S rDNA high-throughput sequencing. The overall experimental framework is summarized in Fig. 1.

### Determination of soil pH and enzyme activities

The soil pH was measured using a pH meter (LPH-A, Labgic, China) with a soil-water ratio of 1:5. Moreover, soil enzyme activities, including sucrase (SUC), urease (UE), and acid phosphatase (ACP), were determined using assay kits from Beijing Solarbio Science and Technology Co., Ltd. (China), with catalog numbers of BC0240, BC0120 and BC0140, respectively. In brief, SUC activity was based on 3,5-dinitrosalicylic acid colorimetry (18); UE assay was determined by the method of indophenol blue colorimetry (19); and ACP assay was performed with the disodium phenyl phosphate colorimetry (20). Absorbance for these assays was read at 540 nm, 630 nm, and 660 nm, respectively.

### DNA extraction and sequencing analysis

The bacterial DNA from 0.25 g of soil was processed using MOBIO PowerSoil kit (MOBIO, CA, USA), and the DNA concentration and purity were checked by NanoDrop One (Thermo Fisher Scientific, MA, USA). Then, DNA quality was analyzed using 1% agarose gel electrophoresis. To identify bacterial community, the 16S rDNA primers (V3–V4 regions) 338F: 5′-ACTCCTACGGGAGGCAGCAG-3′ and 806R: 5′-GGACTACHVGGGTWTCTAAT-3′ were used (21). The PCR reactions were conducted as follows: 94°C for 5 min; followed by 30 cycles of 30 s at 94°C, 30 s at 52°C, 30 s at 72°C, and a final extension at 72°C for 10 min using BioRad S1000 (Bio-Rad Laboratory, CA, USA). The PCR products were purified by an AxyPrep DNA Gel Extraction Kit (Axygen Biosciences, CA, USA), and the purified products were quantified via the QuantiFluor-ST (Progema, WI, USA). All purified PCR products were used for high-throughput sequencing with Illumina MiSeq platform at the Guangdong Magigene Biotechnology Co., Ltd. (Guangzhou, China).

Raw paired-end reads were first quality-filtered using fastp (version 0.14.1, https://github.com/OpenGene/fastp) with a sliding window approach (-W 4 -M 20). Primer sequences were removed using cutadapt (https://github.com/marcelm/cutadapt/) according to the sequence at the ends of the forward and reverse primer information, resulting in high-quality, primer-free paired-end clean reads. Subsequently, paired-end reads were merged using USEARCH (version 10, http://www.drive5.com/usearch/) with the -fastq_mergepairs function. Default parameters were used, including a minimum overlap length of 16 bp and a maximum mismatch allowance of 5 bp within the overlapping region. A second round of quality filtering was performed on the raw tags using fastp (with the same sliding window parameters: -W 4 -M 20) to obtain high-quality merged reads, referred to as clean tags. Sequences annotated as chloroplasts or mitochondria, as well as representative sequences that could not be classified at the kingdom level, were removed. The resulting high-quality sequences from each sample were retained for downstream analysis, and a comprehensive taxonomy table was generated, including the taxonomic assignments of representative sequences. Consequently, the high-quality reads with >97% sequence similarity were classified into different operational taxonomic units (OTUs) using UPARSE.

### Analysis of bacterial community diversity

OTUs were used to compare similarities and differences in bacterial communities. Shared and unique OTUs among sampling sites and soil layers were visualized using Venn diagrams. Alpha diversity (Shannon and Simpson indexes) was calculated based on OTU tables, and beta diversity was assessed using non-metric multidimensional scaling (NMDS) based on Bray-Curtis dissimilarity using the “vegan” package in R. Redundancy analysis (RDA) of bacterial communities in relation to soil pH and enzyme activities, and correlation heatmap between specific bacterial taxa and soil pH and enzyme activities, were performed using the “vegan” package in R. Bacterial functional profiles were predicted using the PICRUSt2 pipeline. OTU abundance tables were normalized against the Kyoto Encyclopedia of Genes and Genomes (KEGG) and the Clusters of Orthologous Groups (COGs) databases. KEGG Orthology identifiers were summarized into pathway-level functions, and COG family information was obtained by mapping Greengenes IDs to the COG database. Functional annotations were retrieved from the eggNOG database. All sequence-based analyses were performed on the Magigene cloud platform (cloud.magigene.com).

### Identification of uric acid-degrading bacteria

Mixed soil samples (0.5 g) were suspended in PBS buffer (10 mL), then serially diluted to concentrations ranging from 10^−1^ to 10^−6^ and plated on mineral agar medium with uric acid as the sole carbon source (uric acid: 2 g, K_2_HPO_4_: 13.75 g, KH_2_PO_4_: 4.5 g, [NH_4_]_2_SO_4_: 2 g, MgSO_4_·7H_2_O: 0.2 g, CaCl_2_: 0.01 g, FeSO_4_: 0.009 g, MnCl_2_: 0.002 g, agar: 15 g, and distilled water: 1,000 mL). The pH of the medium was adjusted to 7.0 ± 0.1 prior to autoclaving. After 7 days of incubation at 28℃, bacterial colonies were purified based on their morphology. The 16S rDNA region of the purified cultures was amplified using the primers 27F (5′-AGAGTTTGATCCTGGCTC-3′) and 1492R (5′-CGGCTACCTTGTTACGACTT-3′). The quality of the amplified sequences was assessed by agarose gel electrophoresis and sequenced by Hainan Nanshan Biotechnology Co., LTD, China. The uric acid degradation ability of each isolate was evaluated by measuring the diameter of the transparent halo formed on uric acid mineral agar after 36 h of incubation at 28℃. For each isolate, five independent plates were prepared (*n* = 5), and the halo diameter on each plate was measured using a ruler with millimeter precision.

### Statistical analysis

Soil pH, enzyme activities, and the diameter of the transparent halo were analyzed in SPSS (version 25.0). Significant differences were calculated by one-way analysis of variance (ANOVA) followed by least significant difference post-hoc tests at *P* < 0.05. Microbial diversity analyses were conducted in R (version 4.1.1). Comparisons of alpha diversity among sampling sites or soil layers were performed using the Kruskal-Wallis test. Beta diversity differences were evaluated using PERMANOVA based on Bray-Curtis dissimilarity matrices. Heatmap analyses were conducted using Spearman correlation coefficients. Co-occurrence network analysis of the top 30 phylum-level taxa was constructed using Spearman correlation coefficients (|*r*| ≥ 0.5).

## RESULTS

### Soil pH and enzyme activities

Analysis of soil pH revealed a decreasing trend with soil depth under the banyan trees, while pH levels remained relatively consistent in the bamboo forest soil (Table 1). However, the pH differences across soil layers were not significant in all of the sites. The soil enzyme activities (UE, ACP, and SUC) across all sites showed a similar pattern, with a significant decrease as soil depth increased; and the enzyme activities at 20–30 cm soil layer were significantly lower than those of 0–10 cm soil layers (*P* < 0.05).

**TABLE 1:** Soil pH and enzyme activities of different sampling sites and soil layers^*a*^

Sites	Soil layer (cm)	pH	UE (U g^−1^)	ACP (10^3^ U g^−1^)	SUC (U g^−1^)
WF1	0–10	5.32 ± 0.45a	836.42 ± 110.43a	51.97 ± 2.38a	28.49 ± 3.49a
	10–20	4.92 ± 0.62a	772.01 ± 103.24a	31.90 ± 4.52b	20.84 ± 3.28b
	20–30	4.87 ± 0.13a	558.91 ± 75.45b	28.89 ± 2.77b	18.81 ± 2.98b
WF2	0–10	5.22 ± 0.37a	953.30 ± 138.54a	57.78 ± 2.21a	28.05 ± 2.57a
	10–20	5.21 ± 0.40a	935.81 ± 138.61a	32.24 ± 5.97b	20.60 ± 2.69b
	20–30	5.01 ± 0.28a	647.17 ± 92.15b	26.23 ± 4.74b	11.91 ± 2.94c
XF1	0–10	5.72 ± 0.34a	1,570.34 ± 197.81a	45.58 ± 4.17a	30.28 ± 4.06a
	10–20	5.71 ± 0.16a	1,168.79 ± 1,14.71b	42.68 ± 5.55a	26.59 ± 4.39a
	20–30	5.52 ± 0.24a	890.49 ± 131.03b	30.66 ± 4.11b	17.46 ± 2.46b
XB1	0–10	5.51 ± 0.11a	857.09 ± 100.48a	40.71 ± 4.57a	27.17 ± 3.32a
	10–20	5.59 ± 0.14a	832.44 ± 152.82a	17.05 ± 2.67b	16.88 ± 2.94b
	20–30	5.52 ± 0.17a	539.03 ± 126.44b	13.68 ± 4.09b	15.12 ± 2.59b
XB2	0–10	5.49 ± 0.14a	1,127.44 ± 165.42a	45.81 ± 4.25a	25.92 ± 3.18a
	10–20	5.63 ± 0.34a	484.96 ± 50.28b	18.31 ± 2.58b	18.44 ± 3.10b
	20–30	5.45 ± 0.41a	499.27 ± 37.89b	14.90 ± 2.77b	18.61 ± 1.91b

^*a*^ Different lowercase letters indicate significant differences between the same sampling site with different soil layers, determined by one-way ANOVA (*P* < 0.05).

### Analysis of bacterial community characterization

**Fig 2:**
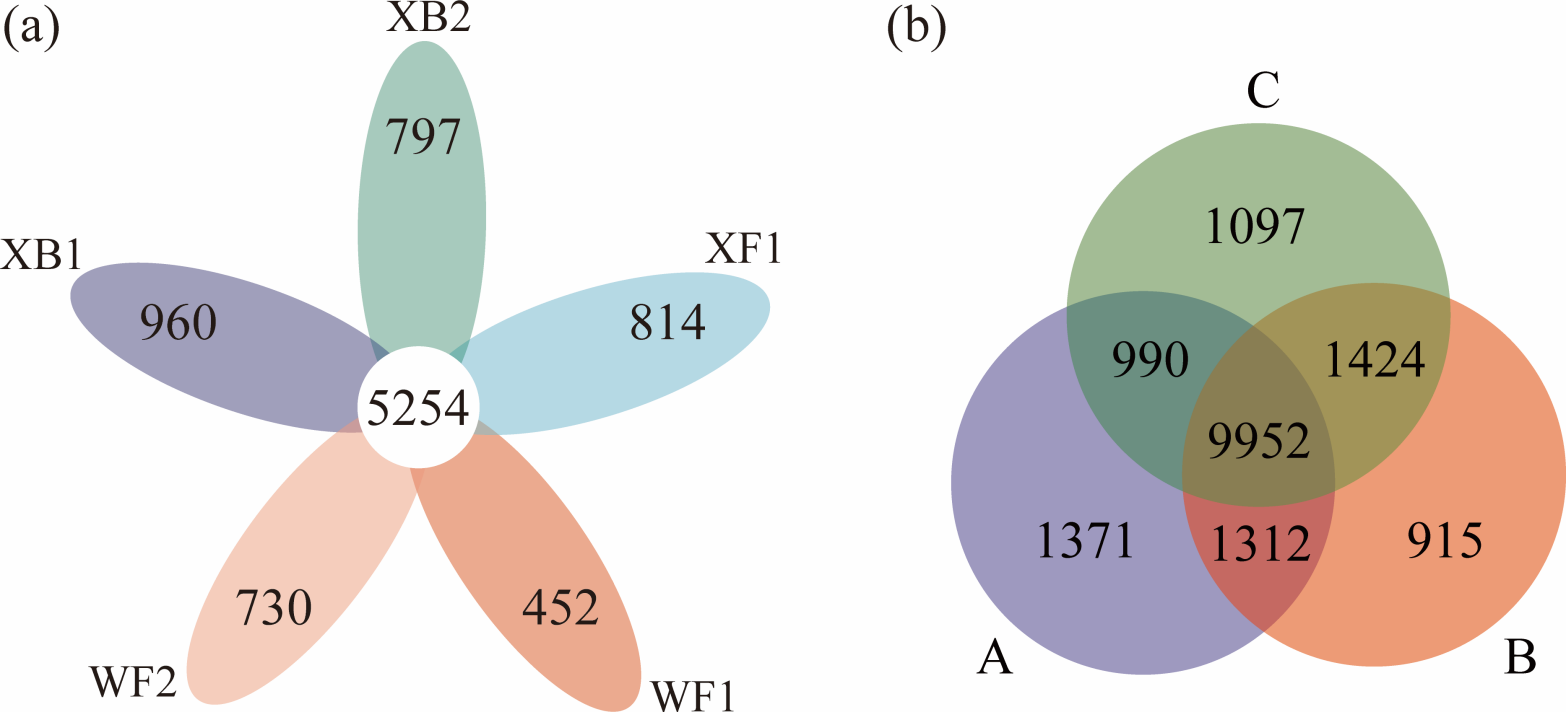
Venn diagrams showing the overlap of bacterial OTUs among sampling sites and soil layers. (**a**) Shared and unique OTUs among the five sampling sites (*n* = 9 per site, total *n* = 45). (**b**) Shared and unique OTUs across the three soil layers, A: 0–10 cm, B: 10–20 cm, and C: 20–30 cm (*n* = 15 per layer).

To investigate the characteristics of soil bacterial diversity in response to a long-term egret colony, five sites were selected and spatially identified. 16S rDNA of 45 soil samples was sequenced, and 5,011,763 raw reads and 17,061 OTUs were obtained (Data S1). In particular, there are 5,254 OTUs shared by all sites, and WF1, WF2, XF1, XB1, and XB2 contain 452, 730, 814, 960, and 797 unique OTUs, respectively (Fig. 2a). Notably, significant differences in OTUs were observed among the three soil layers, with layers A (0–10 cm), B (10–20 cm), and C (20–30 cm) containing 1,371, 915, and 1,097 unique OTUs, respectively (Fig. 2b). These findings suggest that the spatial distribution of soil bacterial communities may play a crucial role in ecological functions.

**Fig 3:**
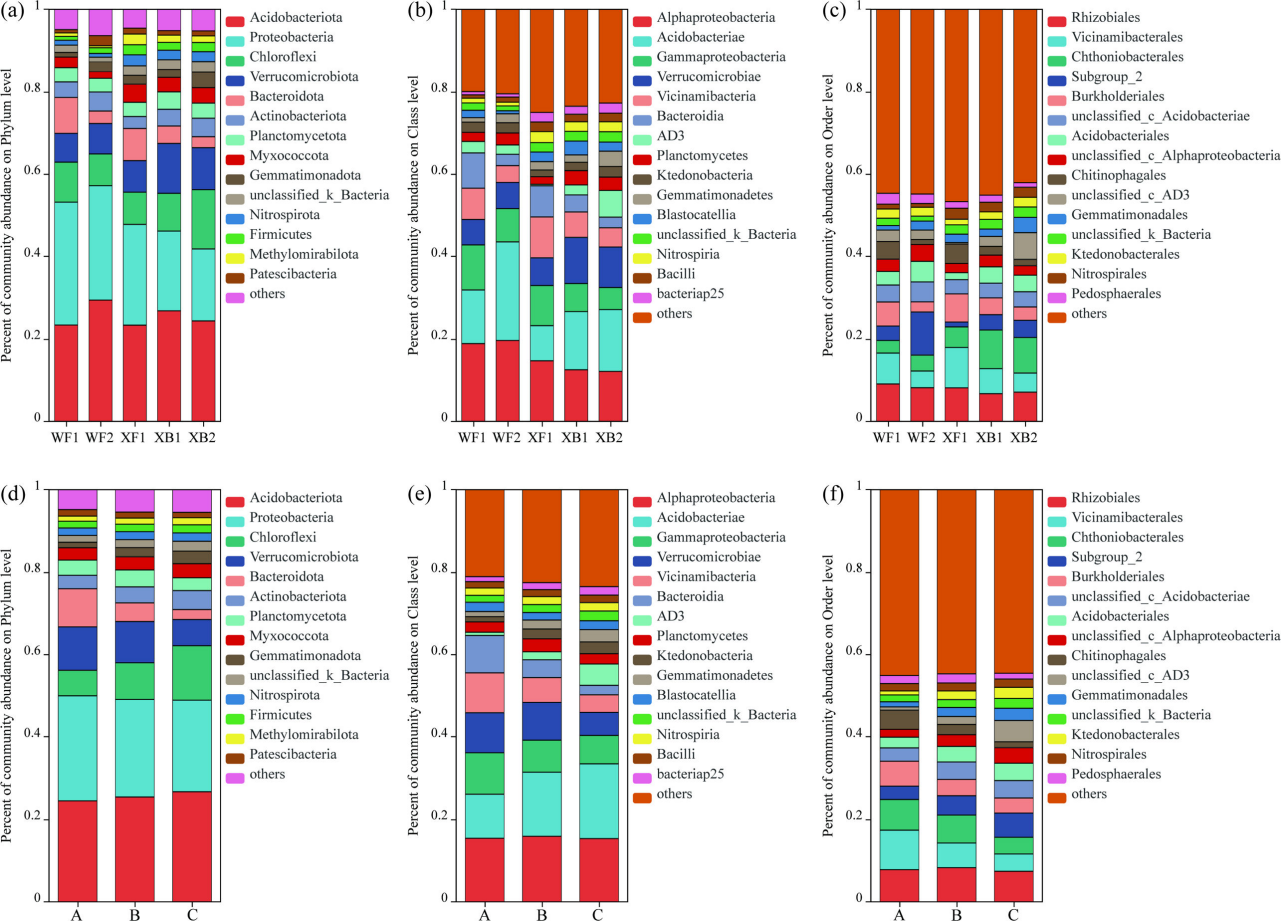
Comparison of bacterial community composition among sampling sites and soil layers. (**a–c**) Bacterial community composition across the five sampling sites and the phylum (**a**), class (**b**), and order (**c**) levels (*n* = 9 per site, total *n* = 45). (**d–f**) Bacterial community composition across the three soil layers at the phylum (**d**), class (**e**), and order (**f**) levels. A, 0–10 cm; B, 10–20 cm; and C, 20–30 cm (*n* = 15 per layer).

Additionally, the relative abundance of bacterial communities at the phylum, class, and order levels was analyzed for the five sites (Fig. 3a through c) and three soil layers (Fig. 3d through f). Across all sites, Acidobacteriota, Proteobacteria, and Chloroflexi were the most abundant phyla, collectively comprising over 50% of the bacterial community. At the class level, Alphaproteobacteria, Acidobacteriae, and Gammaproteobacteria were dominant, accounting for more than 30% of the community. At the order level, Rhizobiales was the most prevalent, followed by Vicinamibacterales and Chthoniobacterales. Additionally, significant differences in bacterial abundance were observed among the three soil layers. At the phylum level, Chloroflexi increased with soil depth (A, 6.18%; B, 8.88%; and C, 13.19%), while Verrucomicrobiota (A, 10.53%; B, 10.02%; and C, 6.35%) and *Bacteroidota* (A, 9.26%; B, 4.50%; and C, 2.42%) decreased significantly. Moreover, Acidobacteriae and AD3 showed a significant decrease with depth, as did Gammaproteobacteria, Verrucomicrobia, Vicinamicrobia, and Bacteroidia. At the order level, the relative abundances of Vicinamibacterales (A, 9.57%; C, 4.20%), Chthoniobacterales (A, 7.45%; C, 4.03%), and Burkholderiales (A, 6.04%; C, 3.64%) were lower in deeper soil layers compared to surface soils.

### Bacterial community diversity

**Fig 4:**
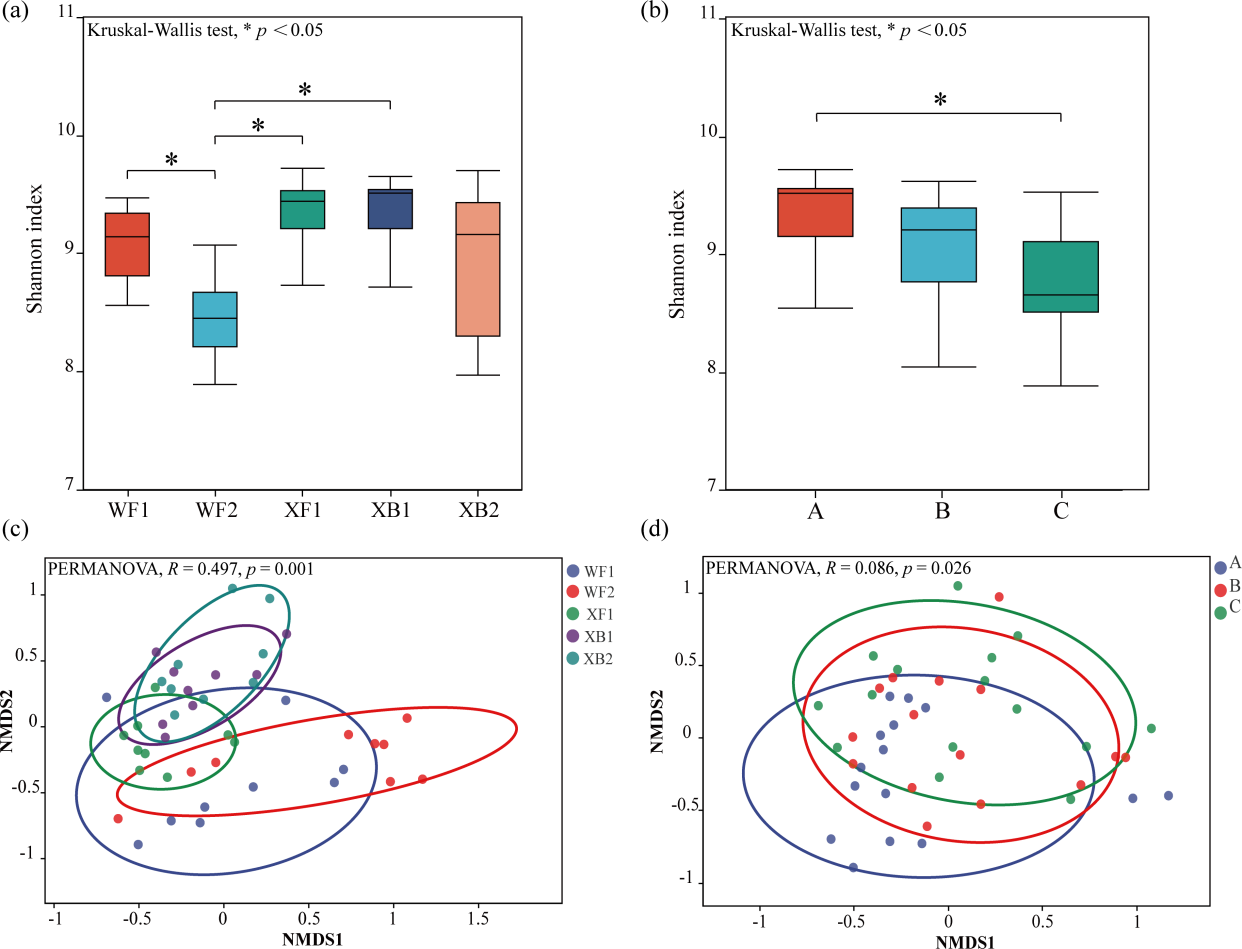
Alpha and beta diversity of bacterial communities across sampling sites and soil layers. (**a and b**) Alpha diversity (Shannon index) across the five sampling sites (**a**) and the three soil layers (**b**), evaluated using the Kruskal-Wallis test (*n* = 9 per site, total *n* = 45). (**c and d**) Beta diversity based on NMDS ordination using Bray-Curtis dissimilarity matrix across the five sampling sites (**c**) and the three soil layers (**d**), with significance assessed by PERMANOVA. “*” indicates a significant difference at the *P <* 0.05. A, 0–10 cm; B, 10–20 cm; and C, 20–30 cm (*n* = 15 per layer).

Significant differences in soil microbial alpha and beta diversity were determined among the five sites and three soil layers (Fig. 4). Among them, WF2 showed the lowest Shannon index, which was significantly different from WF1, XF1, and XB1 (*P* < 0.05; Fig. 4a). In addition, the Shannon index of soil layer A was significantly higher than that of soil layer C (*P* < 0.05; Fig. 4b). Furthermore, comparisons of the Shannon and Simpson indexes across three soil layers within each site showed that soil layer A also exhibited a higher Shannon index and a lower Simpson index than soil layer C (Fig. S1). NMDS further revealed significant differences in the bacterial communities of five sites and three soil layers (*P* < 0.05; Fig. 4c and d). These findings collectively illustrate that bacterial community diversity varies significantly among tree species, across soil layers, and even among sites with the same tree species.

### Relationship between bacterial community characteristics and soil properties

**Fig 5:**
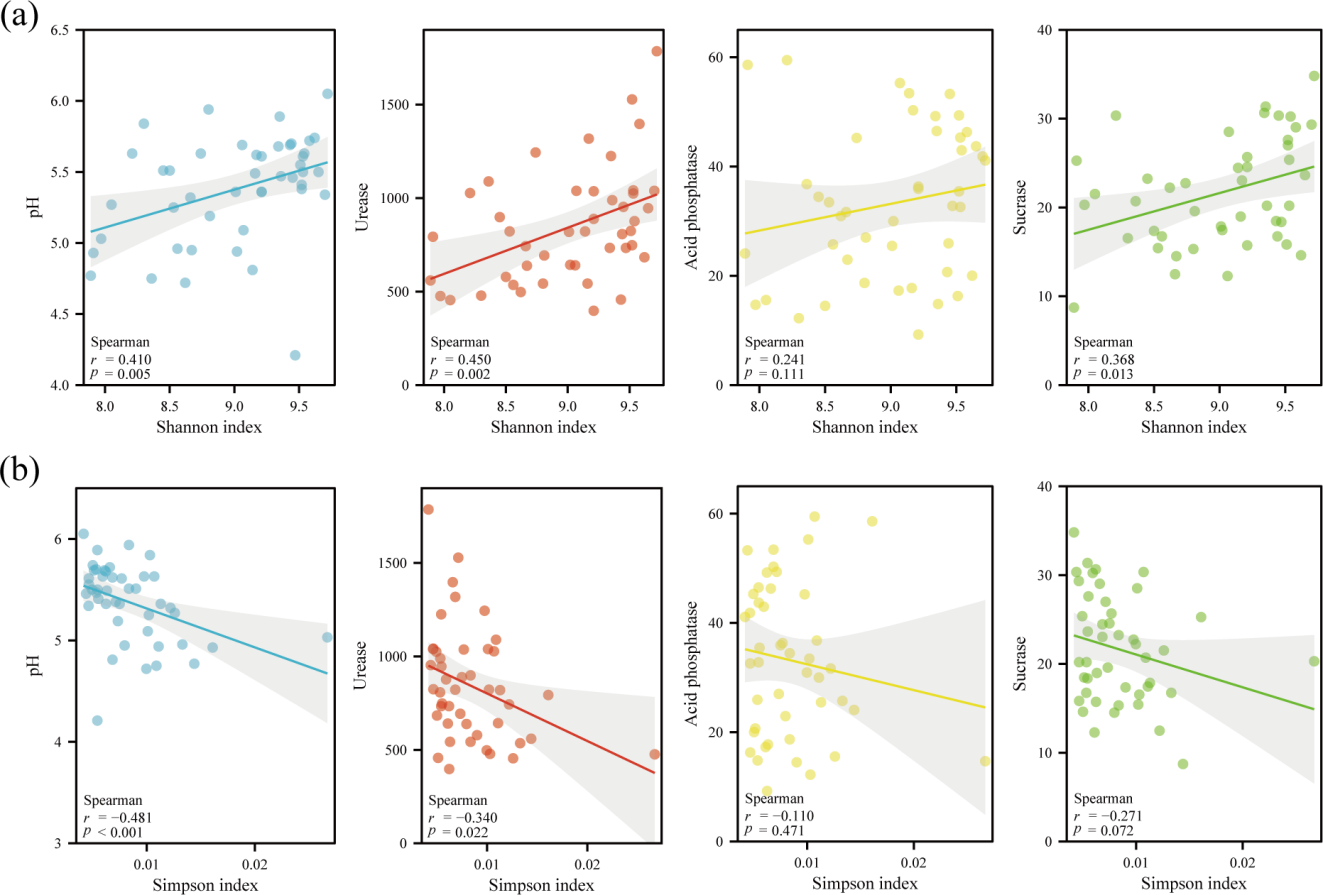
Relationships between alpha diversity indexes and soil physicochemical properties. (**a and b**) Relationships of the Shannon index (**a**) and Simpson index (**b**) with soil pH and enzyme activities, evaluated using Spearman correlation analysis. The solid line indicates the fitted regression trend, and the shaded area represents the 95% confidence interval (*n* = 45).

The bacterial alpha diversity (Shannon and Simpson indexes) exhibited significant association with pH and enzyme activities (Fig. 5). Particularly, the Shannon index positively correlated with pH (*r* = 0.410, *P* < 0.01), UE (*r* = 0.450, *P* < 0.01), and SUC (*r* = 0.368, *P* < 0.05), whereas the Simpson index had negative correlations with pH *(r* = −0.481, *P* < 0.001) and UE (*r* = −0.340, *P* = 0.022). No significant correlations were found between either index and ACP.

**Fig 6:**
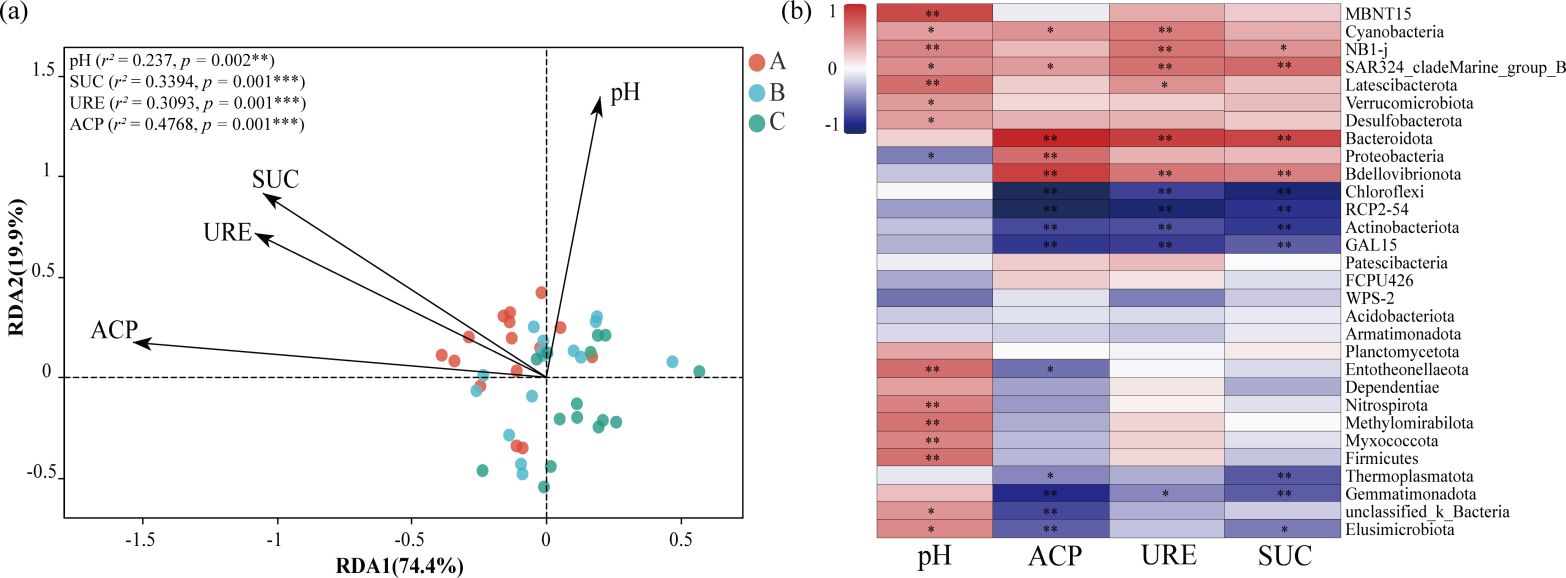
RDA of bacterial communities in relation to soil pH and enzyme activities (**a**) and correlation heatmap between specific bacterial taxa and soil pH and enzyme activities (**b**). The RDA displays all individual samples (*n* = 45), with samples colored by soil layers (A, 0–10 cm; B, 10–20 cm; and C, 20–30 cm; *n* = 15 per layer). The correlation heap map shows Spearman correlation coefficients calculated across all samples (*n* = 45). “*,” “**,” and “***” indicate significance at *P <* 0.05, *P* < 0.01, and *P* < 0.001 level, respectively.

The first axis of the RDA model explained 74.4% of the variance in soil properties affecting the bacterial community, while the second axis accounted for 19.9% (Fig. 6a). These results indicate that soil enzyme activities are positively associated with the soil microbial community, while pH has an opposing effect. Furthermore, the correlation analysis between bacteria taxa and soil environmental factors revealed that Bacteroidota, Bdellovibrionota, and SAR324_cladeMarine_group_B were significantly positively correlated with UE, ACP, and SUC, whereas Chloroflexi, RCP2-54, Actinobacteriota, GAL15, and Gemmatimonadota showed negative correlations with these enzymes (*P* < 0.05 or *P* < 0.01; Fig. 6b). Additionally, MBNT15, NB1-j, Latescibacterota, Entotheonellaeota, Nitrospirota, Methylomirabilota, Myxococcota, and Firmicutes were significantly positively correlated with pH (*P* < 0.01).

### Bacterial correlation network and predictive analysis of bacterial community function

**Fig 7:**
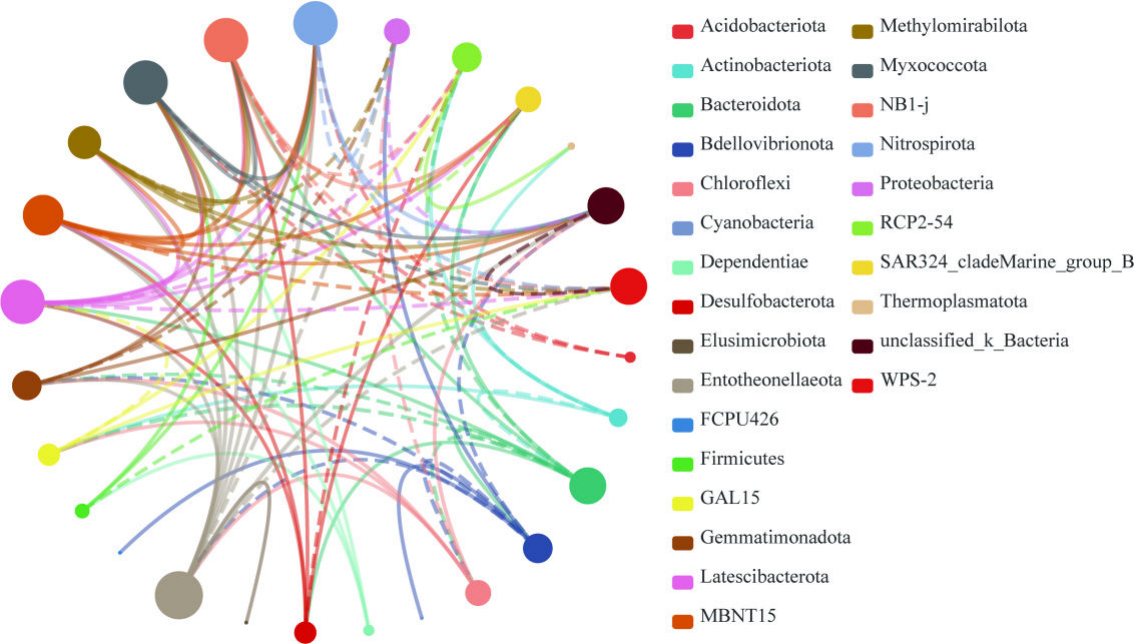
Bacterial interaction network analysis at the phylum level across all soil samples (*n* = 45). Nodes represent bacterial taxon, with node size proportional to the number of connections. Node colors indicate different taxonomic classifications. Edges represent correlations between taxa using Spearman correlation coefficients |*r*| ≥ 0.5, where solid lines indicate positive correlations, and dashed lines indicate negative correlations.

In order to reveal potential co-existence patterns among microbial taxa in response to the egret nesting colony, a co-occurrence network was constructed (Fig. 7). The network analysis revealed extensive interactions across the identified phyla. Here, the top 10 phyla were selected for further analysis, with Entotheonellaeota showing the highest number of correlations (13 lines), followed by Latescibacterota (12 lines), Myxococcota (12 lines), NB1-j (12 lines), Nitrospirota (12 lines), MBNT15 (11 lines), Bacteroidota (10 lines), unclassified_k_Bacteria (10 lines), WPS-2 (11 lines), and Methylomirabilota (9 lines).

**Fig 8:**
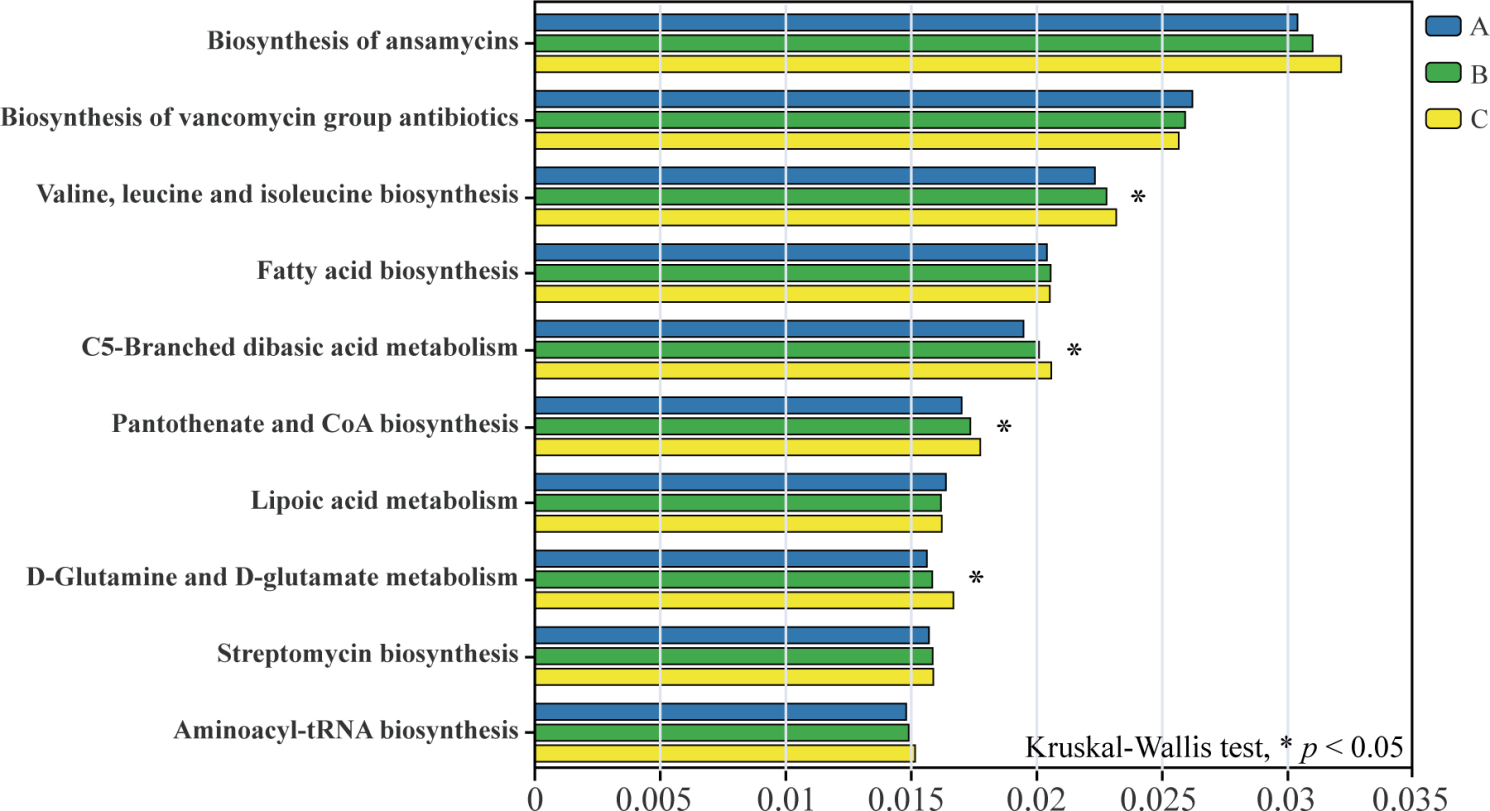
KEGG function prediction of bacterial communities across different soil layers. “*” indicates significant differences among the three soil layers, determined using the Kruskal-Wallis test followed by Dunn’s post-hoc test (*P <* 0.05). A, 0–10 cm; B, 10–20 cm; and C, 20–30 cm (*n* = 15 per layer).

To elucidate the functional potential of the bacterial communities, we performed KEGG pathway prediction (Fig. 8). A clear and significant trend emerged: the relative abundance of pathways related to biosynthesis increased with soil depth. Notably, pathways such as *valine*, *leucine*, *and isoleucine biosynthesis*, *C5-branched dibasic acid metabolism*, and *pantothenate and CoA biosynthesis* were significantly enriched in the deeper soil layers compared to the surface (*P <* 0.05). This trend was further corroborated by COG functional analysis (Fig. S2), which showed that categories including *amino acid transport* and *metabolism* and *coenzyme transport* and *metabolism* were also more abundant in deeper soil. Taken together, these results point toward a fundamental shift in the metabolic strategies of microbial communities along the soil depth gradient.

### Identification of uric acid degradation bacteria

**Fig 9:**
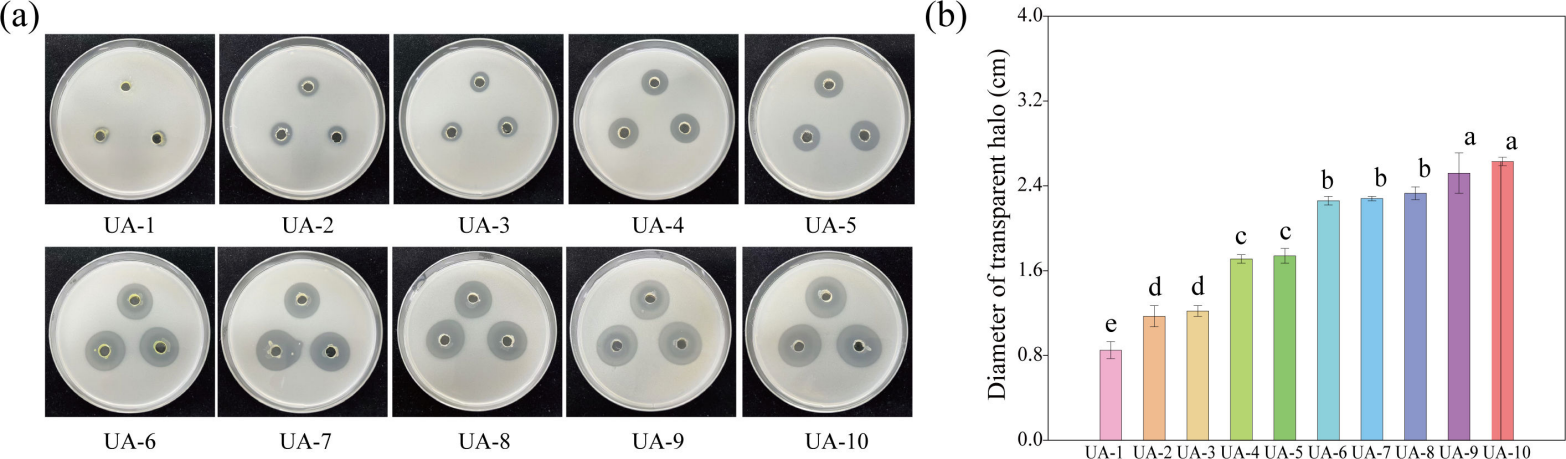
Identification and screening of bacterial strains for uric acid degradation. (**a**) Representative images of bacterial colonies grown on uric acid mineral agar plates. (**b**) Quantification of the diameter of the transparent halo produced by uric acid degradation for each bacterial strain. Each strain was tested in five independent replicates (*n* = 5). Different lowercase letters indicate significant differences among strains, determined by one-way ANOVA (*P <* 0.05). Bacterial strains: UA-1, *Shigella* sp.; UA-2, *Streptomyces* sp.; UA-3, *Escherichia* sp.; UA-4, *Streptomyces* sp.; UA-5, *Streptomyces* sp.; UA-6, *Cellulosimicrobium* sp.; UA-7, *Paracoccus* sp.; UA-8, *Arthrobacter* sp.; UA-9, *Paenibacillus* sp.; and UA-10, *Arthrobacter* sp.

A total of 10 bacteria strains were isolated and ranked based on their ability to decompose uric acid (Fig. 9a). According to 16S rRNA sequencing, the strains UA-1 to UA-10 were identified as follows: *Shigella* sp., *Streptomyces* sp., *Escherichia* sp., *Streptomyces* sp., *Streptomyces* sp., *Cellulosimicrobium* sp., *Paracoccus* sp., *Arthrobacter* sp., *Paenibacillus* sp., and *Arthrobacter* sp., respectively. Particularly, UA-6 to UA-10 demonstrated stronger uric acid degradation abilities (Fig. 9b).

## DISCUSSION

### Soil biogeochemical characteristics under egret colonies

Long-term egret colony activities profoundly impact soil biochemistry, creating a distinct vertical stratification that strongly alters the local ecological environment (6). Moreover, the establishment of protected birds can inadvertently lead to land degradation (8). Assessing soil conditions in bird habitats and implementing effective environmental management strategies are, therefore, essential for ecological conservation. Consistent with previous findings (8, 22), the soils in our study were acidic (pH 4.87–5.72), likely due to nitrification and organic acid production from fecal decomposition. Interestingly, we observed a trend of decreasing pH with depth under the banyan tree (WF1, WF2, and XF1), which may be explained by ammonia volatilization at the surface reducing acid accumulation, while ongoing nitrification and decomposition increased acidity in deeper layers (23).

This top-down nutrient influx from fecal and feathers directly fuels bacterial processes, as evidenced by the significantly higher enzyme activities (UE, ACP, and SUC) in the surface (0–10 cm) layer (Table 1). Soil enzymes are vital indicators of ecosystem functioning (24, 25), and their concentration at the surface confirms this layer as a hotspot for nutrient cycling. In contrast, deeper soils, reliant on nutrient infiltration, exhibit lower organic matter input and consequently reduced enzyme activity. This stratification of resources and activity sets the stage for distinct bacterial community structures at different depths.

### Ecological strategy shifts in bacterial communities along the depth gradient

Our results show that this soil stratification drives a clear divergence in bacterial community structure and ecological strategy. The community was dominated by Acidobacteriota, Proteobacteria, and Chloroflexi (Fig. 3a and d), and also presented the relationship between soil bacterial community and enzyme activity. Acidobacteriota is abundant in organic matter-degrading bacteria, which effectively decompose apoplastic organic matter, sustaining a high nutrient supply and carbon levels (26, 27). Additionally, Proteobacteria, known for their exceptional metabolic diversity, play a crucial role in soil nutrient cycling, particularly in organic phosphate solubilization and nitrogen fixation (28, 29). Previous studies have shown that Acidobacteria and Proteobacteria are widely distributed and sensitive to environmental changes during land use transitions, strongly influenced by soil pH, toxic metals, and terminal electron acceptors, making them potential bioindicators of land use change (30). Similarly, studies in forest ecosystems such as the Andes Mountains and in acidified soils after long-term ginseng cultivation have found that the abundance of *Acidobacteriota* and *Proteobacteria* is closely related to pH and organic carbon levels (25, 31). In our study, these two phyla also dominated the microbial communities under egret-associated conditions, suggesting that egret ecological activity may indirectly shape bacterial composition by altering soil properties such as pH and organic matter input. Notably, the abundance of Chloroflexi increased significantly, driven by oxygen availability and carbon source type. The surface soil (0–10 cm), frequently disrupted and enriched with fresh, labile organic matter from guano, favors fast-growing copiotrophic bacteria that thrive in oxic conditions. In contrast, the deeper soil layers are more stable and are likely characterized by lower oxygen availability (microaerophilic or anoxic conditions) and an accumulation of more recalcitrant, complex carbon compounds (e.g., humic substances) that have leached from the surface. Many members of the Chloroflexi phylum are known to grow slowly and are facultative or obligate anaerobes capable of metabolizing complex carbohydrates (32, 33). Therefore, their enrichment in deeper soil suggests an adaptation to this more oligotrophic and oxygen-limited environment, where they can outcompete other bacteria by utilizing less accessible carbon sources.

### Spatial heterogeneity of bacterial diversity and its drivers

Beyond the clear vertical gradient, bacterial diversity also exhibited significant spatial heterogeneity. Microbial diversity analysis revealed that the Shannon index of surface soil was significantly higher than that of deep soil (Fig. 4b and d) and was positively correlated with soil pH, UE, and SUC (Fig. 5a). This positive correlation aligns with our findings of higher enzyme activity in surface soil (0–10 cm; Table 1), suggesting that the richer microbial community directly contributes to the accelerated nutrient turnover observed in this layer. The RDA demonstrated that soil properties explained over 90% of the variation in bacterial community composition (Fig. 6a), highlighting the critical role of soil properties in constructing microbial community characteristics in different soil layers. Interestingly, the nesting tree species of egrets also significantly influenced soil bacterial diversity (Fig. 4a and c). We acknowledge that a key limitation is the inclusion of heterogeneous tree species. While this design reflects the ecological reality of egret nesting preferences, the inherent differences in vegetation could act as a confounding variable. Our beta-diversity results (Fig. 4c), which show significant separation among all sites, support this view. Xun et al. (34) proposed that root exudates directly affect the soil microbiome by recruiting metabolic-related microbiota from the surrounding soil, accounting for 3.5% of rhizosphere microbiome, which may be a key factor driving this phenomenon. Therefore, the observed spatial heterogeneity in bacterial communities likely arises from a combination of both egret activity intensity and site-specific vegetation. Future studies comparing egret-impacted and non-impacted control sites under a single vegetation type would be beneficial to disentangle these variables. The heatmap showed significant positive or negative correlations between soil properties and the dominant bacterial community (Fig. 6b). Among the taxa, SAR324_cladeMarine_group_B exhibited a significant positive correlation with soil pH and enzyme activities. This species, commonly reported in deep-sea habitats and coral ecosystems (35, 36), is likely introduced into the soil via heron feces following predation. Thus, it may serve as a potential biomarker for heron-affected ecosystems. The observed enrichment of biosynthetic pathways in deeper soil (Fig. 8) can be interpreted as a shift in survival strategy. While surface microbes likely utilize complex nutrients directly from the environment, the deeper microbial communities appear to rely more on their own synthetic capabilities to produce essential compounds like amino acids. This highlights a fundamental functional adaptation to the decreasing nutrient gradient with soil depth. To sum up, the distinct bacterial community compositions observed across soil layers further emphasize their differing ecological functions, underscoring the importance of spatial distribution characteristics in studies of bacterial community diversity.

### Isolation and potential application of uric acid-degrading bacteria

The constant influx of bird droppings creates a strong selective pressure for microbes capable of degrading uric acid, a compound that can cause soil acidification and material corrosion (37, 38). By exploiting this natural enrichment, we successfully isolated five bacterial strains (*Cellulosimicrobium* sp., *Paracoccus* sp., two *Arthrobacter* sp., and *Paenibacillus* sp.) with high uric acid degradation efficiency (Fig. 9). The genera identified are well known for their robust metabolic capabilities and roles in bioremediation. For instance, *Arthrobacter* is widely recognized for degrading organic pollutants, such as aromatic compounds and herbicides, as well as its role in heavy metal fixation due to an extensive enzyme repertoire enabling it to break down complex organic matter into simpler, harmless compounds, contributing to soil health restoration (39, 40). *Paneibacillus*, a plant growth-promoting bacterium, activates plant-induced systemic resistance, enhancing plant defenses against pathogens and playing a crucial role in ecosystems and agriculture (41, 42). Similarly, *Paracoccus* is a highly efficient denitrifying bacterium with significant potential for remediating heavy metal pollution. For example, *Paracoccus pantotrophus* can degrade hydrocarbon sulfur compounds, making it a candidate for bioreactor applications (43). The metabolic diversity and ecological functionality of these bacterial strains underlie their effective utilization of uric acid, suggesting their potential in ecological restoration and environmental management.

### Concluding remarks and future perspectives

Taken together, this study reveals that long-term egret nesting creates a distinct vertical stratification in the soil bacterial communities. The surface soil functions as a zone of active nutrient turnover, characterized by higher enzyme activities and bacterial diversity. In contrast, deeper soil communities appear to adapt to lower nutrient availability by shifting their functional potential toward biosynthesis. This spatial differentiation underscores the complex interplay between egret-derived nutrient inputs and the adaptive strategies of soil microbes within island ecosystems.

While our findings provide a clear snapshot of these patterns, we acknowledge that the inclusion of different nesting tree species is a confounding variable. Therefore, future studies should include non-colony control sites to better isolate the specific effects of the egrets. A key practical outcome of our work is the isolation of five bacterial strains with high uric acid-degrading efficiency. Their potential for bioremediation of ornithogenic waste warrants further investigation, representing a promising avenue for developing biotechnological solutions to environmental challenges in such habitats. In conclusion, this work provides a foundational understanding of the microbial ecology in these important bird habitats and identifies key microbial resources for future applied research.

## Data Availability

The raw reads of 16S MiSeq sequencing reads have been deposited in the NCBI Sequence Read Archive under the BioProject accession number PRJNA1196172. In addition, the 16S rDNA sequences of the bacterial strains with high uric acid-degrading efficiency (strains UA-6 to UA-10) have been deposited in GenBank under accession numbers PQ756993–PQ756997.

## SUPPLEMENTAL MATERIAL

Data S1 spectrum.00015-25-s0001.xls OTUs data.

Supplemental figures spectrum.00015-25-s0002.docx Fig. S1 and S2.

ASM does not own the copyrights to Supplemental Material that may be linked to, or accessed through, an article. The authors have granted ASM a non-exclusive, world-wide license to publish the Supplemental Material files. Please contact the corresponding author directly for reuse.
