# Supplementary material for: Spatial characteristics of soil enzyme activity and bacterial diversity in Chinese egret habitat
Source: Microbiol Spectr. 2025 Dec 30;14(2):e00015-25. doi: 10.1128/spectrum.00015-25 (PMC12889158; doi:10.1128/spectrum.00015-25)

**FIG S1** Soil bacterial alpha diversity (Shannon and Simpson indices) across different soil layers of the five sampling sites. A, 0-10 cm; B, 10-20 cm; C, 20-30 cm (*n* = 3 per layer). Significant differences among soil layers were determined using the Kruskal-Wallis test (*p* < 0.05).


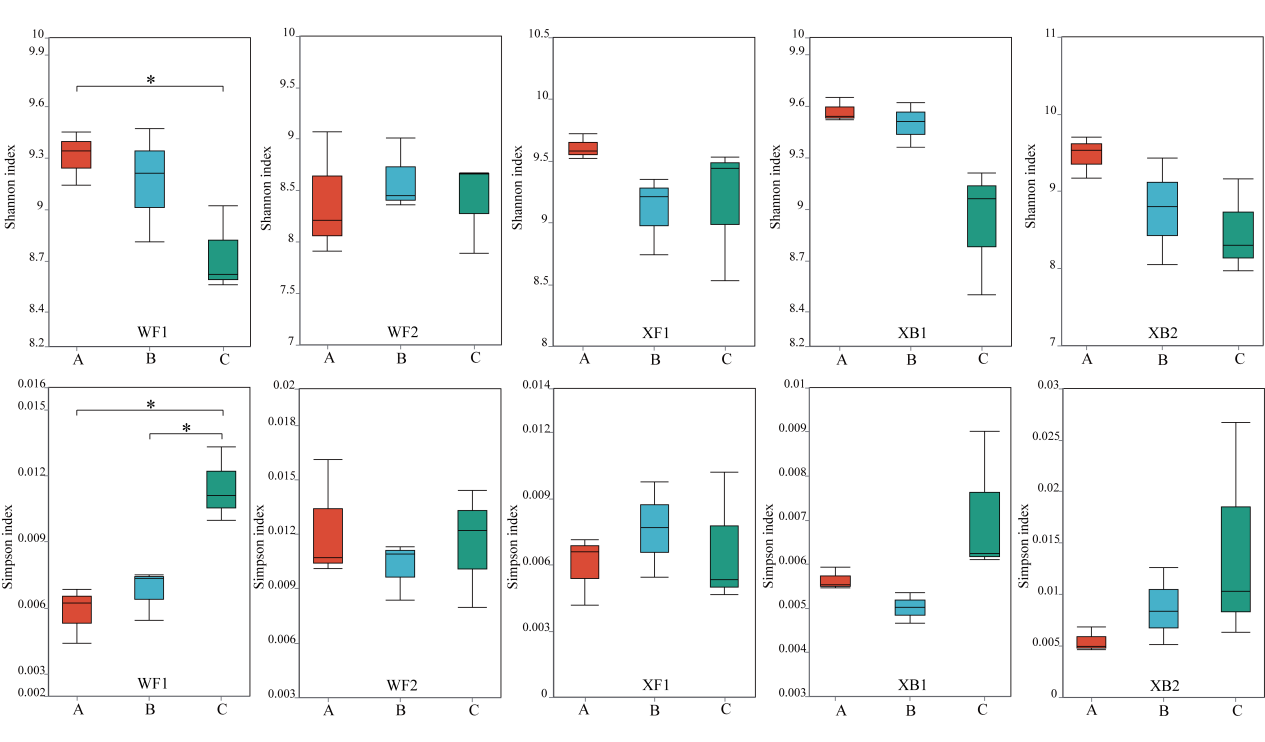


**FIG S2** COG function prediction of bacteria communities across different soil layers. “*” indicates significant differences among the three soil layers, determined using the Kruskal-Wallis test followed by Dunn’s post-hoc test (*p <* 0.05). A, 0-10 cm; B, 10-20 cm; C, 20-30 cm (*n* = 15 per layer).


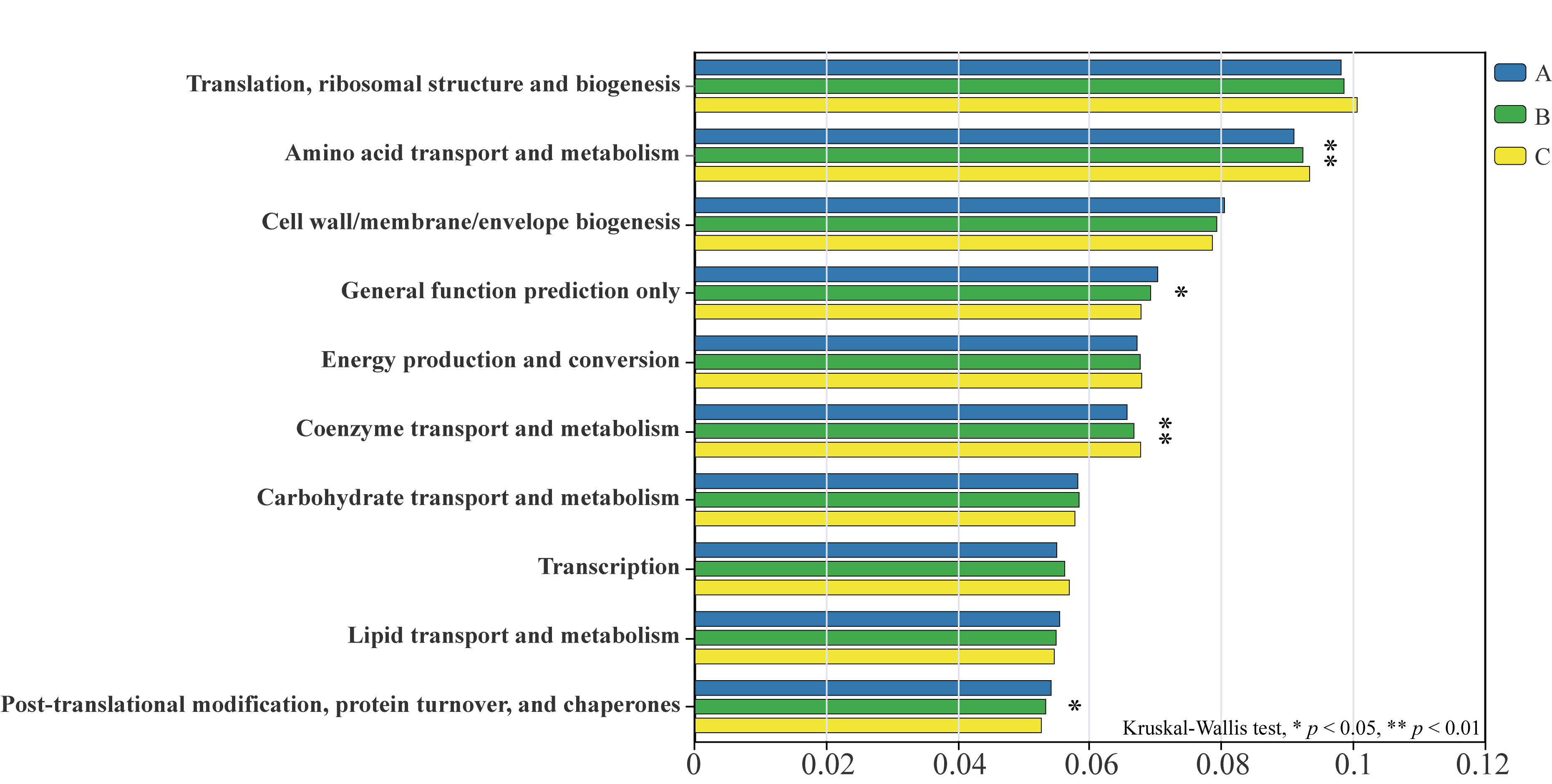

Supplement: Supplemental figures — Fig. S1 and S2. [file spectrum.00015-25-s0002.docx]
